# Contribution of Climate and Air Pollution to Variation in Coronary Heart Disease Mortality Rates in England

**DOI:** 10.1371/journal.pone.0032787

**Published:** 2012-03-12

**Authors:** Peter Scarborough, Steven Allender, Mike Rayner, Michael Goldacre

**Affiliations:** 1 British Heart Foundation Health Promotion Research Group, Department of Public Health, University of Oxford, Headington, Oxford,United Kingdom; 2 Unit of Health Care Epidemiology, Department of Public Health, University of Oxford, Headington, Oxford, United Kingdom; The University of Edinburgh, United Kingdom

## Abstract

There are substantial geographic variations in coronary heart disease (CHD) mortality rates in England that may in part be due to differences in climate and air pollution. An ecological cross-sectional multi-level analysis of male and female CHD mortality rates in all wards in England (1999–2004) was conducted to estimate the relative strength of the association between CHD mortality rates and three aspects of the physical environment - temperature, hours of sunshine and air quality. Models were adjusted for deprivation, an index measuring the healthiness of the lifestyle of populations, and urbanicity. In the fully adjusted model, air quality was not significantly associated with CHD mortality rates, but temperature and sunshine were both significantly negatively associated (p<0.05), suggesting that CHD mortality rates were higher in areas with lower average temperature and hours of sunshine. After adjustment for the unhealthy lifestyle of populations and deprivation, the climate variables explained at least 15% of large scale variation in CHD mortality rates. The results suggest that the climate has a small but significant independent association with CHD mortality rates in England.

## Introduction

Geographical inequalities in coronary heart disease (CHD) mortality rates in England are substantial and persistent. Since the late 1970s, male CHD mortality rates have been at least 30% higher in the North of England than in the South East, and the differences between North and South for female rates have been even larger [1]. Small scale geographic variations also exist, with female mortality rates for CHD in local authorities in the South East of England more than double those of the lowest in the same region, and neighbouring wards within local authorities experiencing CHD mortality rates that are considerably different [2]. If all local authorities shared the same CHD mortality rate as Kensington & Chelsea then there would be over 32,000 fewer deaths from CHD in England every year [1]. The fact that low mortality rates are attained in some areas implies that they are an achievable target with modern standards of prevention and treatment.

It is unclear how much of the geographic variation in CHD in England is a result of differences in the physical environmental. This paper explores the impact of climate and air pollution on geographic variation in CHD mortality rates. Plausible mechanisms for the effect of these factors on CHD have been suggested. Cold weather increases blood pressure, blood cholesterol, blood viscosity (thereby increasing the risk of thrombosis), and could induce a mild inflammatory response thereby increasing blood coagulability [3]. Low exposure to sunlight could increase blood cholesterol levels, since laboratory studies have shown that sunlight is a catalyst for the synthesis of a precursor for cholesterol (squalene) into vitamin D [4]. Exposure to air pollution can provoke an inflammatory response, which increases blood coagulability (and hence risk of thrombosis), the association between air pollution and lung disease could also affect CHD via hypoxia, and air pollution may possibly affect the autonomic nervous system leading to heart rate variability [5]. The temporal influence of climate and air pollution on CHD rates has previously been demonstrated either in time-series analyses [6]–[8] or in seasonal mortality patterns [9], and geographic variation in cardiovascular disease mortality rates in Sheffield [10] and in the US is associated with air pollution [11]. Previous studies that have addressed geographical variations in CHD have either used data on individuals collected from different sites but have been under-powered at the area-level to consider more than one environmental variable simultaneously [12], [13], or have used area-level data and have been unable to adjust analyses adequately for behavioural risk factors for CHD [14], [15]. This paper addresses these gaps in the literature by reporting an analysis of the association between climate and air pollution and CHD mortality rates in a large dataset of small areas, using an area-level measure of the prevalence of behavioural risk factors introduced, that we have previously used to investigate the role of deprivation and unhealthy lifestyle on geographic variations in CHD [16]. The aim of this is to estimate the amount of geographic variation in CHD mortality rates in England that is a result of climate and air pollution after adjustment for the behavioural risk factor profile of populations, deprivation and urbanicity.

## Methods

The analyses reported in this paper utilise ecological regression models, with all standard table wards as the unit of analysis. Standard table wards are a statistical set of boundaries based on the electoral ward boundaries as of 1^st^ January 2003. Henceforth these areas are referred to simply as ‘wards’. There are 7,929 wards in England, which can be grouped into 355 local authorities (LAs). Mortality data were provided by the Office for National Statistics for the years 1999 to 2004 (inclusive) stratified by sex, ward and five year age group. The mortality data included all deaths in England where CHD was recorded as the primary cause of death (for 1999 and 2000, ICD codes 410–414; for 2001–2004, ICD codes I20–25). Change in ICD coding over the data collection period is thought to have had little impact on reporting of CHD mortalities [17]. Rates were constructed using mid-2001 population data stratified by sex, ward and five year age group, collected for the 2001 UK census, and were directly standardised to the European Standard Population.

### Data on the physical environment

Data on mean maximum temperature and total hours of sunshine were provided by the Meteorological Office for 37 English weather stations for every month between 2000 and 2002. The data were used to generate model-based ward-level estimates of mean maximum temperature and total hours of sunshine for each month between 2000 and 2002 using second order trend surface modelling [18], where the climate estimates from the weather stations were used as the dependent variables in a regression model with grid references of the weather stations as the independent variables. The resulting models were used to estimate mean maximum temperature and total hours of sunshine for all wards in England, using the central grid reference for each ward. The modelled monthly estimates were then combined to produce aggregated estimates for the period 2000–2002.

Air pollution data were collected in 2001 for the development of the physical environment domain of the Index of Multiple Deprivation 2004 [19]. The data were drawn from the National Atmospheric Emissions Inventory which estimated annual mean concentrations of benzene, nitrogen dioxide, sulphur dioxide and particulates for all 1 km grid scores within the United Kingdom, using data on location of roads, housing, agriculture and point sources of emissions (e.g. power stations) [20]. These data were used to model estimated annual mean concentrations for each super output area in England. In addition, a single measure – the air quality index – was constructed that is a standardised index of levels of the four pollutants with comparison to recognised safe levels [21]. The air quality index was used in the analyses reported here, after aggregation to ward level by producing averages of the super output area estimates, weighted by population.

### Data on unhealthy lifestyle

An index of unhealthy lifestyle was used as a measure of the behavioural risk factor profile of populations. This index was derived from a principal components analysis of five sets of ward-level synthetic estimates of the prevalence of cardiovascular risk factors, specifically consumption of less than five portions of fruit and vegetables per day [22], body mass index> = 30 kg/m^2^ [22], blood pressure> = 160/95 mmHg [22], blood cholesterol> = 6.5 mmol/l [22], and current smoking [23]. The development of the index of unhealthy lifestyle is described elsewhere [16], and an assessment of the validity of the included synthetic estimates is described elsewhere [24].

### Data on deprivation and urbanicity

Deprivation and urbanicity are other potential confounders of the relationship between climate, air pollution and CHD mortality rates. Deprivation in England is higher in the North than in the South (following a similar gradient to mean temperature and hours of sunshine), and air pollution is higher in more urban areas. The deprivation index used in these analyses was the ward-level Carstairs index [25], generated using data from the 2001 census at ward level [26]. The index is a sum of the z scores of census variables regarding unemployment, overcrowding, car ownership and low social class. The urbanicity variable was a categorisation of all wards into one of three groups: coastal and countryside, urban, and metropolitan. This categorisation was based on the Office for National Statistics area classification variable, which categorises all wards in the United Kingdom into nine supergroups, 17 groups and 27 subgroups, based on a cluster analysis on demographic structure, household composition, housing, socio-economic status, employment, and industry [27]. The categorisation of English wards into the nine supergroups, and then into the urbanicity variable used in this paper, is displayed in table 1.

**Table 1:** Categorisation of English wards (n = 7,932) by the Office for National Statistics (ONS) area classification variable and the urbanicity variable used for this paper.

Urbanicity variable	ONS area classification	Wards (%)	Population (%)
Coastal and countryside	Coastal and countryside	1,838 (23)	8.14M (16)
	Accessible countryside	899 (11)	2.79M (6)
Urban	Industrial hinterlands	1,211 (15)	9.46M (19)
	Traditional manufacturing	524 (7)	4.69M (9)
	Built up areas	163 (2)	0.95M (2)
	Student communities	306 (4)	2.64M (5)
	Suburbs and small towns	2,504 (32)	14.90M (30)
Metropolitan	Prospering metropolitan	169 (2)	1.86M (4)
	Multicultural metropolitan	318 (4)	4.01M (8)

### Statistical techniques

Initially exploratory data analysis techniques were used to investigate correlations between the exposure variables and assess the distribution of the outcome variables. Then baseline multi-level regression models (wards nested in local authorities (LAs)) were built with male and female CHD mortality rates as outcome variables, in order to get a baseline measurement of residual variance at ward-level and LA-level. Then univariate and multivariate multi-level models were built with the physical environment, unhealthy lifestyle index and deprivation variables as exposure variables. Inclusion of variance at ward-level and LA-level is important as climate and air pollution vary on different spatial scales. Finally, equivalent spatial error regression models were built with the same exposure and outcome variables. These were built to assess whether the associations derived in the multi-level models were adversely affected by spatial autocorrelation bias. Results from the multi-level models were the primary outcomes, as they allow for an assessment of how much variance is explained by the exposure variables both at ward-level and LA-level. These results are used as proxies for explanation of ‘small-scale’ variation (e.g. variation in CHD mortality rates within a city) and ‘large-scale’ variation (e.g. variation in CHD mortality rates between regions of England, such as the North and South). The estimation technique used for the multi-level modelling was iterative generalised least squares (IGLS), and the spatial error modelling used maximum likelihood techniques, ensuring that the results of the multi-level models and the spatial error models are comparable.

## Results

Both male and female ward-level age-standardised CHD mortality rates were reasonably normally distributed, and hence suitable for regression analyses. Table 2 shows descriptive statistics for the dependent and independent variables. Eight wards featured zero female CHD deaths in the six year data collection period – these wards were retained in the data analysis as they had little impact on the distribution of the outcome variables. Both maximum temperature and hours of sunshine showed little variance (a range of only 3.2°C and 400 hours of sunshine annually). These two variables were also correlated (r = 0.63), and were negatively correlated with both the unhealthy lifestyle and deprivation indices. Air pollution was significantly higher in more urban areas.

**Table 2:** Summary statistics, correlation co-efficient matrix of the continuous exposure variables, and mean of exposure variables by urbanicity category (wards, n = 7,929).

*Variable*	*Range*	*Interquartile range*	*Standard deviation*	*Mean*	*Median*
CHD mortality rate per 100,000, men	24.4 to 525.3	142.5 to 212.1	53.6	179.9	174.9
CHD mortality rate per 100,000, women	0.0 to 336.2	63.0 to 100.6	29.7	83.6	80.5
Mean max. temp (°C)	11.2 to 14.4	13.5 to 14.4	0.6	13.9	14.1
Sunshine (000s hrs/yr)	1.3 to 1.7	1.4 to 1.6	0.1	1.5	1.5
Air quality index (SDs)	0.4 to 2.2	0.9 to 1.3	0.3	1.1	1.1
Unhealthy lifestyle, men (SDs)	−6.7 to 5.3	−1.2 to 1.2	1.8	0.0	−0.1
Unhealthy lifestyle, women (SDs)	−6.2 to 5.6	−1.3 to 1.3	1.8	0.0	−0.1
Deprivation (SDs)	−5.7 to 16.5	−5.4 to 15.1	3.5	−0.1	−1.0

	*Coastal & countryside (n, %)*	*Urban (n, %)*	*Metropolitan (n, %)*
Urbanicity	2737, 35%	4708, 59%	484, 6%

Correlation co-efficient matrix						
	Mean max. temp	Sunshine	Air quality index	Unhealthy lifestyle, men	Unhealthy lifestyle, women	Deprivation
Mean max. temp	1.00					
Sunshine	0.63	1.00				
Air quality index	0.21	−0.07	1.00			
Unhealthy lifestyle, men	−0.43	−0.40	−0.07	1.00		
Unhealthy lifestyle, women	−0.44	−0.39	−0.12	0.99	1.00	
Deprivation	−0.19	−0.17	0.42	0.57	0.51	1.00

Mean of continuous exposure variables by urbanicity category				
*Variable*	*Coastal & countryside*	*Urban*	*Metropolitan*	*p for trend*
Mean max. temp (°C)	13.8	14.0	14.7	<0.001
Sunshine (000s hrs/yr)	1.5	1.5	1.6	<0.001
Air quality index (SDs)	0.9	1.2	1.6	<0.001
Unhealthy lifestyle, men (SDs)	−0.1	0.2	−1.4	0.001
Unhealthy lifestyle, women (SDs)	−0.1	0.3	−2.1	<0.001
Deprivation (SDs)	−1.8	0.3	6.0	<0.001

*SDs = Standard Deviations.*

The ward-level and LA-level variance in the baseline models is shown in table 3. For men, 73% of the total geographic variation in CHD mortality rates was at ward-level, and 74% of the total geographic variation in female CHD mortality rates was at ward-level, with the remainder at local authority-level. This suggests that the average variance in CHD rates for wards within a local authority was three times higher than the variance between local authorities within England, and hence that small scale geographic variations in CHD rates are larger than large scale geographic variations. Univariate analyses (models A, B and C, table 4) showed that each of the exposure variables were strongly associated with both male and female CHD mortality rates. In both male and female multivariate models, the beta coefficient for sunshine was strongly attenuated when included alongside temperature and air pollution (model D, table 4). The multivariate models containing only climate and air pollution variables explained a considerable amount of variance at LA-level (56% and 60% in male and female mortality models, respectively) but very little of the ward-level variance.

**Table 3:** Residual variance at ward-level (n = 7,929) and local authority-level (n = 354) for baseline (no exposure variables) and final models (MODEL L).

		BASELINE		FINAL	
		Variance	Standard Error	Variance	Standard Error
MEN	Ward-level	2,096.4	34.1	1,580.2	25.7
	LA-level	779.7	66.3	166.1	18.1
WOMEN	Ward-level	660.8	10.7	547.6	8.9
	LA-level	226.8	19.5	53.5	6.0

**Table 4:** Beta coefficients for multi-level regression models for physical environment exposure variables in univariate (MODELS A–C) and multivariate (MODEL D) analyses, and after further adjustement for confounding variables (MODELS E–F).

	MODEL A	MODEL B	MODEL C	MODEL D	MODEL E	MODEL F
*Beta coefficients in models for male CHD mortality rates*						
Mean max. temp (°C)	−27.7**			−32.7**		−12.5**
Sunshine (000s hrs/yr)		−162.6**		−18.2		−27.3*
Air quality index (SDs)			54.4**	56.6**		5.8
Urban†					2.0	1.9
Metropolitan†					−5.9	−8.0*
Unhealthy lifestyles (SDs)					6.5**	5.0**
Deprivation (SDs)					7.2**	7.2**
Ward-level variance explained	0%	0%	3%	3%	25%	25%
LA-level variance explained	43%	34%	−8%	56%	68%	79%
*Beta coefficients in models for female CHD mortality rates*						
Mean max. temp (°C)	−15.5**			−17.2**		−7.9**
Sunshine (000s hrs/yr)		−90.2**		−14.2		−14.3*
Air quality index (SDs)			23.7**	26.5**		5.7**
Urban†					0.5	0.4
Metropolitan†					2.9	1.2
Unhealthy lifestyles (SDs)					4.1**	3.3**
Deprivation (SDs)					3.0**	3.0**
Ward-level variance explained	0%	0%	2%	2%	17%	17%
LA-level variance explained	45%	35%	−2%	60%	62%	76%

*SDs – Standard Deviations;*

† *in comparison to coastal and countryside wards;*

* *significant at p<0.05;*

** *significant at p<0.01.*

Table 4 also shows the results for the multi-level model that includes all of the exposure and confounding variables (model F). Nearly 80% of LA-level variance in both male and female CHD mortality rates was explained, and around 20% of the ward-level variance. Beta coefficients for the climate and air pollution variables were heavily attenuated after inclusion of the confounding variables. The maximum temperature variable showed a significant negative association with both male and female CHD rates after adjustment for deprivation, urbanicity and unhealthy lifestyle, and sunshine was also independently (though weakly) associated with CHD rates. The air quality index variable showed only a small association with CHD mortality rates after adjustment for confounding variables (this association was non-significant for men).

The physical environment variables contribute little to the explanation of ward-level variation. However, they clearly contribute to the explanation of LA-level variance in mortality, even after adjustment for urbanicity, the unhealthy lifestyle and deprivation indices: the models containing only the confounding variables (model E) explained around 65% of the LA-level variance, whereas this increased to nearly 80% in the final model (model F).

The spatial error univariate and multivariate models showed good agreement with the multi-level models, suggesting that spatial autocorrelation bias has not substantially affected these findings. The parameter estimates in the spatial error models tended to be closer to zero than in the multi-level models, demonstrating that spatial autocorrelation (when unaccounted for) tends to result in a bias away from the null hypothesis. The difference in the parameter estimates between the multi-level and spatial error models was generally in the region of around 10% to 20% (results not shown).

## Discussion

### Statement of principal findings

Two local climate measures (mean daily maximum temperature and total hours of sunshine) and a measure of air pollution were found to explain - without accounting for other factors - nearly 60% of large scale geographic variation in CHD mortality rates but did little to explain small scale geographic variations in CHD rates. The strength of the relationships was strongly attenuated when deprivation, urbanicity and behavioural risk factor profiles of populations were added as explanatory variables. A substantial amount of large scale geographic variation in CHD rates is explained by physical environment variables even after adjustment for deprivation, urbanicity and behavioural risk factor profiles of populations – at least 10% of large scale variation in mortality rates. These models suggest that the climate has a small but independent association with CHD mortality rates in England – a ward with the lowest observed temperature had 40 more male deaths per 100,000 and 25 more female deaths per 100,000 than a ward with highest observed temperature, all else being equal. In comparison, applying excess winter mortality from CHD for England in 2004/05 [9] to temporal differences in temperature in England [28] suggests an increase in CHD mortality of approximately 3 deaths per 100,000 for men and 2 deaths per 100,000 for women. This suggests that the association between climate and CHD mortality rates shown in these analyses may be due to residual confounding, but it should be noted that temporal variations and geographic variations in CHD mortality rates due to temperature are not directly comparable. If environmental exposures contribute more to long-term cumulative risk rather than short-term risk, then it's plausible that geographic variation is indeed a much larger contributor than seasonal variation. The fully adjusted analyses suggest that air pollution has a small association with geographic variation in CHD mortality rates, however this finding may be due to over-adjustment - one of the mechanisms of the impact of urbanicity on health is via air pollution levels. However, without adjusting for urbanicity (such as model D), the association between air pollution and CHD mortality rates may be confounded by other mechanisms for the urban-health relationship, such as access to healthcare. Since the air quality index is a more direct measure of air pollution than the urbanicity variable, the limited association of air pollution with CHD shown in the fully adjusted model seems the most plausible interpretation of these results.

### Strengths and weaknesses of the study

This is the first instance of a study of geographic variation in small area CHD rates that accounts for behavioural risk factor profiles of populations, deprivation, and a number of measures of the physical environment within the same set of analyses. The multi-level design of the analyses allowed for the explanation of large scale and small scale geographic variation in CHD rates simultaneously, which allowed for disentanglement of the influence of variables that are effective at the different scales. The spatial error models allowed for an assessment of whether the multi-level models were prone to spatial autocorrelation bias, which was shown not to be the case. The systematic approach to building models that was utilised here allowed for a comprehensive assessment of the impact of confounding, and for some disentanglement of the amount of geographic variation that is explained by the climate and air pollution variables.

The results presented in this paper are derived from ecological cross-sectional analyses. Because of the cross-sectional nature of the studies, the results cannot confirm causal relationships. The relationship between climate and CHD rates presented here may be a result of residual confounding. Economic deprivation, unhealthy lifestyles and the climate generally follow the same North-South gradient in England, and the associations shown in the analyses may be a result of errors in the measurement of economic deprivation and unhealthy lifestyles, or could be due to unmeasured and potentially confounding factors such as utilisation and quality of health care. A previous study of women in 23 towns in Great Britain suggested that controlling for aspirin and statin use (as a proxy for health service utilisation) removed the residual variance in adjusted cardiovascular prevalence rates in England (but not in Scotland) [13], suggesting that this residual confounding could explain the associations with climate found here. However, the longitudinal impact of climate on CHD mortality rates is well established, so a potential impact of climate on CHD mortality rates is plausible. The ecological nature of the study design implies that the results cannot provide any information about how the explanatory variables affect individuals [29]. For example, the results imply that the average temperature of an area has an impact on CHD rates within that area, but they do not tell us anything directly about how the temperature of an area affects the individuals living in the area, or whether certain individuals within the area are more at risk than others. Risk factors for CHD tend to accrue over the life course [30], and so examining a cross-sectional relationship with the physical environment and unhealthy lifestyle will tend to under estimate the impact of these variables. This is particularly problematic as the analyses did not take account of migration between wards in England. The exposure and the confounding variables used in these analyses are derived from a number of different data sources and using different techniques. It is therefore difficult to assess the degree of uncertainty in the results that is due to measurement error, but this is likely to have had some impact on the results.

### Comparison with other studies

The results presented here are in general agreement with the UK literature on geographic variation in CHD rates, in that not all of the variation in CHD rates can be explained by lifestyle factors alone. The British Regional Heart Study (BRHS) provides the most comparable results for the impact of climate on geographic variation in heart disease in England, despite the widely differing methodology employed in the study compared with the analyses reported here. Analysis of phase one of the BRHS (which utilised ecological analyses of CHD mortality rates in 253 towns) suggested that in 1969–73 climate variables had a modest effect on variation in local CHD mortality rates after adjustment for deprivation [14], which is a similar result to those reported here. Phase two of the study (a cohort study of 7735 men in 24 British towns, followed up for fifteen years) showed that temperature explains around 30% of the between-towns variance in CHD incidence rates that remained after adjustment for social class and individual-level risk factors [12]. Again, this is in broad agreement with the results reported here – that the climate has a modest effect on CHD rates after adjustment for differences in the behavioural risk factor profile of populations and socio-economic status.

The results of this paper extend the results of phase one of the BRHS in the following ways: all wards in England were included in the analysis; a measure of the behavioural risk factor profile of populations of areas was included; an exploration of both small scale and large scale geographic variation in CHD rates was conducted; including wards from rural areas allowed for urbanicity to be included as a potential explanatory variable; more sophisticated estimates of air pollution and climate were used, which allowed for modelled estimates of these measures to be applied to all wards in England. The results of this paper complement the results of phase two of the BRHS, but extend the interpretations to women and to men of all ages. In addition, the analyses reported here were sufficiently powered at the area-level to allow for inclusion of several environmental variables in the models simultaneously.

The results presented here suggest that air pollution has a small positive association with CHD mortality rates in small areas. A similar finding was shown by Maheswaran et al. in an analysis of census enumeration districts in Sheffield [10], where nitrogen oxide levels were significantly associated with increased CHD mortality rates (smaller, non-significant associations were also shown for carbon monoxide and particulates). Interestingly, the Sheffield analysis showed no association between air pollution and CHD hospital admissions, suggesting that air pollution may increase the risk of sudden death from CHD (although residual confounding could not be ruled out). Secondary analysis of a cohort study restricted to US metropolitan areas with estimates of particulate air pollution [11] also showed small but significant increases in cardiovascular deaths for residents in areas with increased air pollution (for both current and former smokers).

### Implications and further research

The analyses reported here suggest that, on top of excess winter mortality, CHD mortality rates in the coldest parts of England are generally higher compared to the warmest parts (although this association may be due to residual confounding). Whilst this difference is small compared to differences in the lifestyle of populations, if the relationship is shown to be causal then it is an area which could be targeted in order to reduce geographic inequalities in CHD. Analyses of excess winter mortality in different regions of Europe have shown that the excess mortality is generally greater in countries with milder climates and this has led researchers to suggest that the impact of a cold climate on cardiovascular health can be substantially reduced if the population were better prepared for cold weather by improving household heating and insulation and wearing more appropriate clothing during cold periods of the year [31], [32]. Interventions such as these would be beneficial for reasons other than improving cardiovascular health. Cold weather has been implicated in the development of a number of conditions such as respiratory disease, particularly in elder people. Improvements in home heating have the potential to improve quality of life, and increased insulation of homes would reduce fuel use thereby saving household finances and reducing greenhouse gas emissions. Further research should be conducted to determine cost-effective interventions to reduce the impact of climate on coronary heart disease mortality. Such interventions have the potential to reduce geographic inequalities in health in England. With regard to air pollution, the results of this study are inconclusive as to whether raised levels of air pollution in urban areas lead to increased levels of CHD in comparison to rural areas. This needs further investigation, using more refined small area data of air pollution (preferably directly measured), CHD incidence and confounding variables (e.g. small area prescription rates for aspirins/statins, access to health care etc.), and including small areas drawn from rural and urban areas.

### Prior publication

The work reported in this manuscript has not previously been published elsewhere, or submitted for publication elsewhere.
